# The Diamond Project: A Quality Improvement Model for Adopting Shared Service Delivery in the Washington Vaccines for Children Program

**DOI:** 10.3389/fpubh.2020.00272

**Published:** 2020-07-14

**Authors:** Betty Bekemeier, Kylerose Delaney, Stacy Wenzl, Michele Roberts, Dorene Hersh

**Affiliations:** ^1^School of Public Health, University of Washington, Seattle, WA, United States; ^2^Washington State Department of Children, Youth, and Families, Olympia, WA, United States; ^3^Washington State Department of Health, Olympia, WA, United States; ^4^Public Health – Seattle & King County, Seattle, WA, United States

**Keywords:** vaccines for children, shared-service delivery, cross-jurisdictional sharing, foundational public health services, public health departments

## Abstract

**Context:** Increasing federal requirements with no change in the Centers for Disease Control and Prevention budget creates an unsustainable delivery model between states and their local counterparts for programs like Vaccines for Children (VFC).

**Project:** The Washington State Department of Health collaborated with the Washington Association of Local Public Health Officials to identify how best to improve the quality of the VFC program.

**Approach:** Utilizing Quality improvement and Lean Six Sigma methods, the project team was able to adopt a new shared-service delivery model to improve the quality of the VFC program in Washington State.

**Discussion:** Through utilization of quality improvement methods and Lean methodology Washington State Department of Health identified recommendations to adopt a shared-service delivery and implemented those changes.

## Introduction

Today's public health departments operate in environments of chronic fiscal stress from severe cuts to state and local health jurisdiction (LHJ) budgets (1, 2). This stress exists as they are responsible for responding to urgent public health issues (2–4) and emergencies and addressing “upstream” prevention activities with consistently inadequate resources. At the same time, state departments of health are also faced with new challenges as federal program requirements change or increase expectations without additional funding (5). The Vaccines for Children Program (VFC), a Centers for Disease Control and Prevention (CDC) initiative, is one such program. VFC requires, among other activities (or “Tasks”), that grant awardees evaluate enrolled providers via quality assurance site visits (5). In 2011 the CDC increased the site visit mandate from every 4 to every 2 years—essentially doubling the work of site visit facilitators, without a corresponding funding increase (5). LHJs have also been working to implement new systems and polices to support compliance with other state and federal mandates and many are also pursuing public health accreditation, with its implications for system-level quality improvements. In recent years, many LHJs have been expected to support, and in some communities lead, healthcare transformation efforts arising from the Affordable Care Act (6).

These system-level changes, constrained budgets and limited resources, as well as a desire to assure a focus on improving immunization-related outcomes in line with the foundational public health services provided by public health agencies (4), prompted public health leaders in Washington State to rethink the way VFC services were provided in the state.

Literature suggests that adopting shared-services models, or Cross Jurisdictional Sharing (CJS), can facilitate pooling of resources and talent/capabilities, and can lead to more effective use of funding and service delivery (2, 4). CJS has been used successfully for emergency preparedness services in areas with low financial resources and across multiple jurisdictions (1, 3). In addition, CJS has been used to share programs, services, and staff among smaller LHJs (3). The literature shows that CJS is widely used across the U.S. but there is little information available describing how CJS methods and models are evaluated and adopted by local and state public health authorities (3). CJS can potentially be used more widely across public health programs to improve the quality of services in the face of resource constraints and could ultimately transform how public health agencies ensure a basic level of foundational public health services for every community. Public health professionals could, thus, benefit from having clear examples and documented approaches from which to adapt their practice.

The current literature lacks descriptions of models and frameworks that public health organizations have used to guide evaluation and implementation of CJS. This paper provides a useful example of a project between Washington State Department of Health (DOH) and the Washington State Association of Local Public Health Officials (WSALPHO) that identified and utilized quality improvement (QI) models as a framework to improve their VFC program. QI Six Sigma and Lean methodology, principles, and tools helped guide the system changes.

## Methods

The method used in reconstructing and describing this system change process included content review of materials developed during this 1.5 year-long public health practice-based project and informal interviews with members of the DOH and WSALPHO project team as well as the project facilitators. The interviews and material review were conducted by the second author (Delaney) as part of a graduate degree capstone project. The interviews were conducted by Delaney with the facilitators and members of the project team to identify related materials, determine the flow of events and activities, clarify understanding, and draft an initial comprehensive description of the process. In terms of the materials obtained, compiled, and reviewed; these included facilitation plans, meeting minutes, flow charts, reports, presentations, and communications pertaining to the state's process. Delaney aligned and reviewed documents using a timeline, examining information in chronological order in terms of how the project progressed and as a means to assess what methods were used for each step. Public health practice-based research faculty (Bekemeier) and project team members, representing both state (Roberts) and local (Wenzl and Hersh) public health practice, provided deep background support, validation, oversight, and substantial contributions to this reconstruction of events and this paper's development.

### Context

In 2016, Washington State DOH identified that their approach to delivery of their federally-funded VFC program, was unsustainable. DOH was the grantee of VFC funds and contracted several of the VFC requirements to the 35 LHJs in Washington. Over the years, compliance mandates for VFC program participation increased the number of activities expected of the state (e.g., supply chain and vaccine oversight, quality oversight of providers). These increases, together with stagnant funding amounts, resulted in multiple LHJs dropping out of some VFC service activities or withdrawing from participation in the VFC program altogether. Compounding this, were challenges with maintaining standardization of VFC services across all of Washington's 35 LHJs, leading to variability in service quality and efficiency and related frustrations at the state and local levels. The overall governmental public health role became overly focused on maintaining VFC compliance, rather than improving vaccine uptake—even as Washington's immunization rates lag behind the Healthy People goal of 80% vaccination coverage, with just under 70% of Washington toddler-age children receiving all recommended vaccines (7). State leaders were, thus, increasingly concerned about missed opportunities to support LHJs in focusing on local projects to increase immunization rates. Meanwhile, decreased funding and increased requirements and operating costs had created tension between state and local public health leaders, as well as a lack of trust surrounding fiscal and shared services decisions.

As a means to improve the quality and sustainability of the VFC program in Washington, as well as enhance and improve focus on increasing uptake of vaccines to improve immunization rates, DOH leaders moved to partner with LHJs to explore cross-jurisdictional sharing and a redesign of the VFC service model in Washington State. Quality improvement principles, methods and tools were used to neutralize tensions surrounding cross-jurisdictional sharing and guide objective decision-making regarding the allocation of activities, roles and responsibilities, and consequent reallocation of resources in support of those decisions. These methods have been described in the literature as deeply rooted in business and are generally used to help increase value and eliminate waste to improve program implementation (8). The principles used are (1) focus on the system and the processes of service delivery, (2) utilize a team-based approach, (3) involve staff who do the work in the decision process, (4) use data to drive decisions, and (5) focus on meeting the customer's needs (8, 9). This paper demonstrates how QI and Lean models, can be used to establish sustainable and improved public health systems and services.

### Approach to VFC Program Improvements: Project Initiation

As the VFC grantee, DOH was required to meet VFC requirements for all activities (herein referred to as Tasks) addressed within their federal cooperative agreement. Historically, DOH contracted with Washington's 35 LHJs to complete some of these seven CDC Tasks: (1) Renewing provider agreements (enabling participation in the VFC program and access to vaccines), (2) Enrolling new providers in the program, (3) Provider vaccine ordering, (4) Provider vaccine accountability, (5) Technical assistance and consulting, (6) VFC site visits (quality monitoring and control) and (7) Assessment, Feedback, Incentives Exchange (AFIX) visits (5). By 2017, however, five LHJs had opted out of all contracted VFC program Tasks and another five had opted out of several Tasks, placing additional burden on DOH. DOH was obligated to ensure the work got done, even within counties in which LHJs had dropped out. Additionally, there was little standardization among the LHJs that continued to provide VFC-related Task activities. These Tasks and related processes were variably conducted, inefficiently executed, and competing for resources that could be better utilized promoting immunization uptake in communities.

These factors prompted DOH leadership to revisit VFC service delivery. Given the scope of these problems and the likely impacts to local funding and relationships between and among LHJs, DOH sought input and collaboration from WSALPHO. WSALPHO is a non-profit group of LHJ leaders serving counties across Washington (10). DOH engaged WSALPHO leadership, presenting them with the challenges and inviting them to participate in a collaborative system improvement project. The Diamond Project was, thus, created and comprised of a team of state and local public health staff. Once the partnership between DOH and WSALPHO was established for the VFC quality improvement project, the organizations worked together to assign representative executive sponsors and establish their roles in the effort (11). A team was formed consisting of mid-level managers and supervisors, and front-line staff from both state and local levels of practice.

During the project's first phase, the team created a charter, establishing roles and responsibilities and defining project goals (12). The charter helped establish that DOH and WSALPHO would, together, make recommendations. The charter also established that DOH would have final approval of resulting recommendations, given the state's responsibility as the CDC's VFC grantee. The charter helped establish shared understanding to avoid conflict during decision-making and implementation processes. Establishing executive sponsorship and creation of a charter are basic strategies in both QI and project management sciences (11, 12).

The established team also determined the importance of having QI experts and neutral facilitators involved, leading them to work with Washington State's Public Health Centers for Excellence (CfE) to facilitate the project (13). The CfE is a partnership between two different county-level Washington LHJs that provides consultation, training, and facilitation services (13).

DOH and WSALPHO chose CfE for their expertise in QI, Lean Six Sigma methods, and connection to public health practice. The team was assigned four CfE facilitators who were experts in the methods of QI and Lean. Funding for this work and the facilitators came from Washington's CDC immunization cooperative agreement. CfE facilitators led the team in deciding which QI models and Lean principles would be applied to the project.

Project goals were initially drafted by DOH, then shared with LHJ team members for feedback. The goals then went to CfE facilitators for applying a Lean methodology in further refining them. CfE used background documents and conducted interviews with stakeholders, including other LHJ staff, to establish these project goals:

1. Create a consistent statewide standard of immunization promotion and vaccine compliance services that meet customer needs;
2. Create efficiencies in the services DOH/LHJs provide through consolidated contracts in Washington State; and
3. Identify effective immunization promotion activities to improve immunization rates in Washington State.

The Diamond Project's use of principles from basic QI and Lean Six Sigma, assured a focus on improving the value of services by understanding and meeting users or customer needs, targeting, and eliminating inefficiencies in processes (such as unnecessary waiting or delays and underutilized staff skills), and developing standard approaches to the work. Six Sigma reduces defects in systems or services through problem solving (9). This model has a quantitative data focus that supports QI by helping reduce the influence of emotions and personal relationships in driving decisions (9).

**Figure 1:**
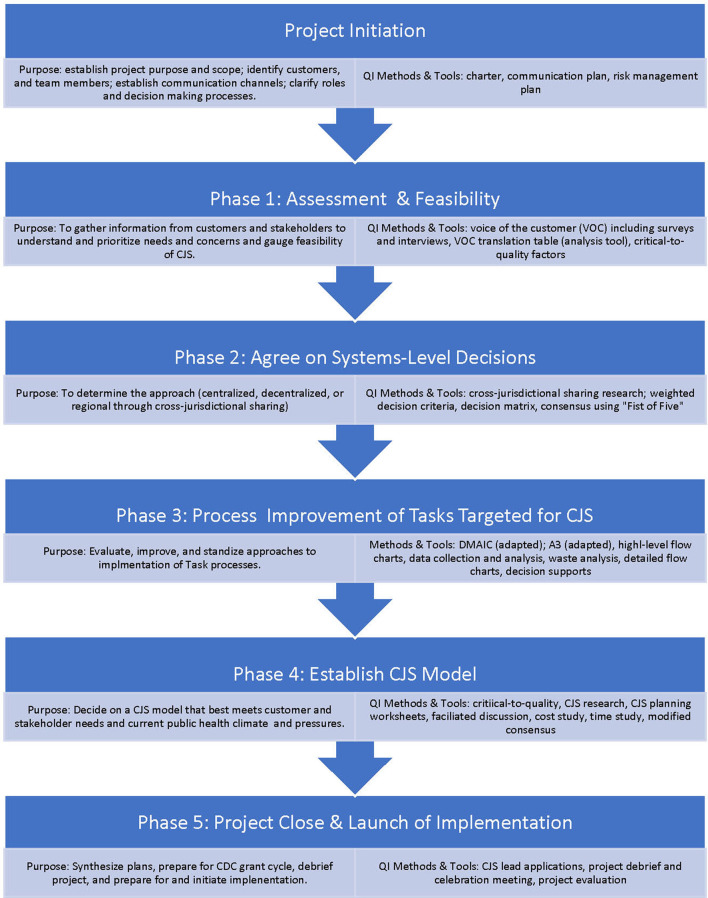
Overall Diamond Project phases.

Once sponsors, team members and facilitators were chosen, they collaboratively created a communication and risk management plan to help manage the change effort and mitigate tensions surrounding cross-jurisdictional sharing (Appendix A in Supplementary Material). The plan was developed at the beginning of the effort but updated and utilized continuously over the course of the project. Because the team consisted of individuals across Washington, team meetings were conducted via conference calls and three in-person multi-day meetings over the 2-year project. In addition, team members engaged other LHJ staff and leadership stakeholders through email and in-person meetings to obtain their feedback on findings and decisions discussed during The Diamond Project team meetings. After initial scoping and chartering, the project was launched and executed using a six-phased approach. Figure 1 depicts the project flow by phase, each phase's purpose, and a sample of QI tools used in each phase.

### Assessment and Feasibility

The project's first phase focused on assessment of customer needs, referred to as Voice of the Customer (VOC) in Lean Six Sigma. VOC is used to identify user's or customer's expectations and needs, and in this project helped determine the feasibility of a CJS approach (14). The assessment targeted three stakeholder or customer groups: LHJ immunization staff, LHJ leadership, and healthcare providers enrolled in the VFC program (i.e., direct customers). Assessment data from these three groups helped identify needs of healthcare providers and provided perspectives and rationale to guide the consideration and exploration of systems-level redesign, considering three modes for delivery of services: centralization of Task activities, decentralization of Task activities and CJS approaches. The assessment data included surveys and interviews conducted with local clinical providers in the VFC program and with LHJ staff. Additionally, project team feedback and LHJ communications between team members were recorded and reviewed, as a means to gain a better understanding of Washington's existing VFC program. Key findings emerged from the assessment for each of the three stakeholder groups. From these findings, project sponsors and facilitators concluded that there was sufficient interest and a willingness to consider CJS approaches, and the project team proceeded to develop recommendations for systems-level changes in the state VFC program administration.

### Systems-Level Recommendations

With findings from the assessment in hand, the team commenced with exploration and analysis of the seven Tasks to determine the systems-level at which each of the Tasks should be executed—centrally, de-centrally, or through CJS. For each of the three models, the project team developed guiding criteria by which to evaluate the Tasks. For example, one of the criteria adopted for CJS was “geographic proximity”—or when efficiencies in service delivery can be achieved because of geography, such as when onsite services are required. The criteria under which each approach was evaluated is found in Appendix B in Supplementary Material. The criteria were weighted and each of the Tasks were evaluated by the project team against the weighted criteria associated with each of the three systems-level approaches being considered, using a decision matrix (Table 1). A decision matrix is a QI tool for identifying options, developing objective criteria for decision-making, and applying criteria to the options (15). Agreement on criteria weights, and on the scoring of the Tasks against the criteria was achieved through discussion and group consensus. At the conclusion of the discussion, each Task received one overall score for each of the three models considered. After final review and deliberation, the team concluded that the highest score among each of the approaches (a score for each centralized, decentralized, or CJS approach) and for each Task, determined the “best” delivery model for each of the seven Tasks (Table 2) and The Diamond Project team's recommendations.

**Table 1:** Decision Matrix with weighted scores.

				**Task 1: renewing provider agreements**		**Task 2: enrolling new providers**		**Task 3: provider vaccine ordering**		**Task 4: provider vaccine accountability**		**Task 5: provider TA**		**Task 6: provider site visits**		**Task 7: AFIX**	
		**Criteria explanation**	**Criteria Wt**	**Score**	**Wt score**	**Score**	**Wt score**	**Score**	**Wt score**	**Score**	**Wt score**	**Score**	**Wt score**	**Score**	**Wt score**	**Score**	**Wt score**
Criteria set: DOH as the “WHO”	1	Highly specialized expertise is needed	0.17	10	1.65	0	0.00	10	1.65	10	1.65	10	1.65	10	1.65	10	1.65
	2	Work can be done completely remotely/automated	0.09	10	0.92	−10	−0.92	10	0.92	10	0.92	0	0.00	−10	−0.92	0	0.00
	3	Economies of scale	0.13	10	1.32	0	0.00	10	1.32	10	1.32	10	1.32	0	0.00	0	0.00
	4	High overhead/capital casts	0.39	10	3.95	−10	−3.95	10	3.95	10	3.95	10	3.95	10	3.95	10	3.95
	5	High need for standardization	0.18	10	1.78	10	1.78	10	1.78	10	1.78	10	1.78	10	1.78	10	1.78
	*e*	LHJ leadership input re effectiveness [survey data}	0.04	0.385	0.01	−2.69	−0.10	0.769	0.03	−0.385	−0.01	−1.154	−0.04	−2.308	−0.09	−2.8	−0.11
Wt total					9.63		−3.19		9.65		9.61		8.65		6.37		7.28
Task rank					2.00		7.00		1.00		3.00		4.00		6.00		5.00
Criteria set: each LHJ as the “WHO”	1	Geographic proximity needed	0.09	−10	−0.92	0	0.00	−10	−0.92	−10	−0.92	0	0.00	10	0.92	0	0
	2	Attention to local needs/tailoring needed [i.e. specialized interventions}	0.25	−10	−2.51	−10	−2.51	0	0.00	−10	−2.51	−10	−2 51	10	2.51	10	2.51
	3	Need relationship building/community trust	0.36	−10	−3.62	10	3.62	0	0.00	0	0.00	10	3.62	10	3.62	10	3.62
	4	Local knowledge is needed (politics, needs, partner landscape etc.)	0.17	−10	−1.70	0	0.00	0	0.00	−10	−1.70	0	0.00	0	0.00	10	1.70
	5	Beneficial to have local jurisdictional authoritative presence	0.10	−10	−1.03	−10	−1.03	−10	−1.03	−10	−1.03	−10	−1.03	−10	−1.03	−10	−1.03
	6	LHJ leadership input re effectiveness (survey data)	0.02	3.846	0.09	5	0.11	2.308	0.05	3.077	0.07	−4.615	0.10	6.538	0.15	6	0.13
Wt Total					−9.69		0.19		−1.90		−6.09		0.19		6.16		6.93
Task Rank					7.00		4.00		5.00		6.00		3.00		2.00		1.00
Criteria set: LHJ collaboratives as the “WHO”	1	Shared priorities/common goals	0.13	−10	−1.29	0	0.00	−10	−1.29	−10	−1.29	10	1.29	0	0.00	10	1.29
	2	When it makes geographic sense/proximity	0.04	−10	−0.41	10	0.41	−10	−0.41	−10	−0.41	0	0.00	10	0.41	10	0.41
	3	Opportunities to leverage partnerships	0.13	−10	−1.29	10	1.29	−10	−1.29	0	0.00	10	1.29	0	0.00	10	1.29
	4	Local cross-jurisdictional expertise	0.20	−10	−2.04	10	2.04	−10	2.04	10	2.04	10	2.04	10	2.04	10	2.04
	5	Economies of scale	0.41	10	4.08	10	4.08	10	4.08	10	4.08	10	4.08	10	4.08	10	4.08
	6	LHJ leadership input re effectiveness (survey data)	0.09	−2.96	−0.27	−2.96	−0.27	−1.481	−0.13	−1.481	−0.13	−1.481	−0.13	−1.481	−0.13	−1.154	−0.10
Wt Total					−1.21		7.55		−1.07		4.29		8.56		6.40		9.00
Task Rank					7.00		3.00		6.00		5.00		2.00		4.00		1.00

**Table 2:** Final recommendations by each of the seven Tasks.

**Task**	**Delivery model for task**	**Key rationale**
Task 1: Renewing Provider Agreements	Centralize with DOH	• *Overwhelmingly* the highest scoring option
Task 2: Enrolling New Providers	Establish Cross-Jurisdictional Sharing Model	• CJS was the highest scoring option • Requires a site visit/need for geographic proximity • High potential for economies of scale & increased efficiencies • LHJs still need to maintain relationships
Task 3: Provider Vaccine Ordering	Centralize with DOH	• *Overwhelmingly* the highest scoring option
Task 4: Provider Vaccine Accountability	Centralize with DOH	• *Overwhelmingly* the highest scoring option
Task 5: Technical Assistance & Consulting	Centralize with DOH	• DOH was the highest scoring option • High degree of waste and duplication of efforts currently • This Task closely tied to Tasks 3 and 4; need to align • Great potential for economies of scale (high expertise; existing help desk, existing Immunization Information System)
Task 6: VFC Site Visits	Establish Cross-Jurisdictional Sharing Model	• CJS was the highest scoring option • Lots of inefficiencies in existing model and a high need to improve standardization • High potential for economies of scale (fewer staff, less training expense) • LHJs still need to maintain relationships
Task 7: AFIX Visits	Establish Cross-Jurisdictional Sharing Model **with caveats** (more reservations associated with this recommendation)	• CJS was the highest scoring option • There is a high need for tailoring of services provided under this Task description, which will be closely tied to the evidence-based strategies under this recommendation • Local knowledge is needed • Opportunity for both economies of scale through CJS while also maintaining need for LHJs to maintain relationships with providers

The team then collected feedback regarding these Recommendations from stakeholders that would be effected by changes to the recommended delivery model for each Task. This included vetting recommendations with the broader WSALPHO membership of LHJs outside of the team. This was done via email, group meetings, and peer-to-peer conversations as directed by the communication plan (Appendix A in Supplementary Material). Team members also engaged with leadership and staff at their own LHJs to get input on the proposed recommendations. In addition, meetings of the full WSALPHO membership included discussions about The Diamond Project, including discussion of the recommendations. All feedback was compiled by CfE facilitators and presented to the team. The team used both the decision matrix and stakeholder feedback to finalize recommendations for each Task (Table 2). As established by the charter, DOH made the final decision in January 2017 about what delivery models the Tasks and immunization promotion strategies would use.

The recommendations shifted the delivery model for three of the Tasks to a CJS shared-services model. In addition, four Tasks were shifted from LHJ responsibilities to a DOH responsibility. This concluded Phase 2 of the project and the team then focused on process improvement of the three Tasks targeted for CJS and creation of a plan to implement the changes (Figure 1).

### Process Improvement

Since a new shared-service model was being adopted, the team prioritized development of a CJS plan for the three Tasks approved for CJS (Table 2), first focusing on identifying the process improvements that would be needed to make this change. Given the substantial variability in how the work had historically been done across the state, the team needed tools and supports to ensure standard implementation across all LHJs that assured consistency, reduced duplication, and avoided unnecessary costs. To establish this quality across LHJs, a subgroup of the team developed the scope of improvements needed for each of these Tasks approved for CJS, using project definitions and high-level process flow charts and then proceeded, using Six Sigma process improvement methods. An example of the scope and flow chart for Task 6 is at Appendix C in Supplementary Material.

The Diamond Project team also reviewed the VOC findings and other data, and translated the information into factors that were critical to quality (15). Identifying critical to quality factors is a QI approach in which VOC data are reviewed and analyzed to identify factors most critical to the quality of activities carried out in the system. An example factor identified from interviews with LHJ staff as critical to quality included covering costs or, more precisely, the need to determine how the costs of site visits would be covered in a shared-service model.

Once critical to quality factors were established, the team created a very detailed process flow chart for each Task as a means to eliminate waste and inefficiencies identified in existing processes, while ensuring that customer and stakeholder needs were addressed. An example of this for Task 7 can be found in Appendix D in Supplementary Material. A list of tools or supports for standardizing the new processes was also created for each Task. For example, standard communications with health care providers was identified as a necessary support. At the end of this phase, the team had developed flow charts for processes leading to the ideal state for each of the three Tasks identified for CJS.

### Development of the CJS Model

The next phase was to identify the approach to CJS by which to implement the newly designed processes for Tasks 3, 6, and 7. The goal was to develop efficient, standardized practices and sharing across LHJ county lines that would reduce the number of LHJs carrying out VFC Tasks. Through another QI activity facilitated by the CfE, The Diamond Project team developed several alternative approaches to CJS. Again, through facilitated discussion, the group collectively evaluated several CJS approaches, considering the factors that were critical to quality and the process flows charts. The team ultimately selected a final CJS approach to vet by LHJ leadership in the state. The following recommendations for approaches to CJS were made to LHJ administrators regarding VFC Tasks 3, 6, and 7:

1. Adopt the nine Accountable Communities of Health (ACH) regions in the state as the VFC shared-services regions (Washington's ACHs are the state's structure of regional, multi-sectoral formal collaboratives guiding health and healthcare transformations) (16).
2. Empower each of the nine ACH regions in the state to identify their lead LHJ collaboratively. If a region is unable to reach consensus, DOH staff select the lead through an application process.
3. Empower each region to determine how the LHJ lead can best represent and serve all LHJs within that region.

This CJS approach above called for creation of a nine-region shared-services model, after vetting by other LHJ staff and leadership, and was adopted by DOH in July 2017. The team then gathered additional data to identify the estimated costs of this CJS approach. Two related primary data collection efforts were implemented toward this effort: a time study and a cost survey of LHJs.

The time study was conducted to identify how long, on average, a VFC site visit and an AFIX visit each took, as a means to gauge their actual costs. Using the high-level flow charts of the process for site visits and AFIX visits developed in Phase 3 (Appendix C in Supplementary Material), the team developed a data collection tool to track time for each step in the visit process. Team members tracked their time for a period of 3 months and found that on average, a site visit took 3.47 h, and an AFIX visit took 5.56 h. Historically, LHJs were reimbursed for 10 h per visit.

A cost survey was conducted to gather ideas, concerns, and information on LHJ costs; to establish a more precise cost model than what was historically used to guide VFC funding. DOH used these data to develop a cost model. From the time study and cost survey data collected, the team determined that the new model could save the system ~$500,000 that could be redirected from vaccine accountability tasks, to services that promoted immunization uptake in communities.

Each of the nine regions of multiple LHJs also needed to collaboratively identify the lead LHJ for their region. To facilitate this, DOH created a web page describing a funding opportunity for LHJs to apply for to be the lead regional representative. Nine LHJs (one in each region) submitted applications. Each was assessed by a DOH review panel and was approved to serve as the regional lead.

### Implementation

The final phases of the project coincided with the upcoming CDC grant cycle, allowing for the adopted and substantial changes to be included in the federal grant application. DOH finalized plans in preparation for the CDC grant cycle, including work toward implementation of the new processes and the supports needed to sustain improvements and standardize processes. Due to the changes, new contracts were created between the state and all 35 LHJs. The work of The Diamond Project team concluded with a final debriefing and project closing celebration in March 2018. The CJS model was launched in August 2018.

## Discussion

The Diamond Project has implications for public health policy and practice. The project demonstrates an innovative and collaborative statewide approach to develop and implement systems-level improvements to a crucial statewide public health program. A similar approach can be used to improve VFC program implementation in other states and along with other foundational public health services. While QI and Lean models are deeply rooted in business, The Diamond Project also demonstrates how these principles and models can be adapted for public health and valuable in developing a shared-services approach. These QI and Lean models make effective use of collaborative, data-driven decision-making and likely have promise for re-envisioning other large scale programs and CJS strategies.

Public health is a multi-player system with representation from federal, state, and local levels-all with differing priorities. Yet even as state health departments are often the recipients of federal funding, Washington's Diamond Project demonstrates the value of thoughtful partnering between a state and LHJs in the planning and implementation process for making state-wide program changes. Creating a project team inclusive of local partners acknowledged differing priorities, built rapport among those impacted, and mitigated tensions between state-level administrators and LHJ staff. Use of QI tools, and objective, iterative decision-making processes enabled stakeholders with sometimes competing perspectives to maintain objectivity and a focus on the public health mission and the established shared goals as guideposts for the team. The final implementation plan demonstrated that these public health professionals could work well together to consolidate services and Tasks to create a value-driven system.

When faced with an unsustainable system for immunization services, as federal requirements changed and funding was limited, CJS was identified, through a systematic and collaborative process, as key to helping improve workflow between Washington DOH and its local counterparts. Using QI methods and Lean methodology, The Diamond Project successfully developed recommendations for improvement and implemented those changes, identifying additional resources for improving immunization rates. The projects successes suggest that a shared-services delivery model might be appropriately considered by other states as an efficient and effective approach to improving vaccine delivery services, across health jurisdictions.

## Conclusion

Through adaptations of QI and Lean Six Sigma models, DOH and its LHJ partners were able to make significant changes to the administration of their VFC program. Utilizing QI and Lean methods to support data-driven decision-making and collaborative planning, this approach provides a rich example of applying these methods in a public health setting. Through a systematic decision-making process that involved both the state- and local-levels of the VFC program, Washington's state and local partners made vital large-scale system changes, restructuring and adopting a new shared-service model. This process has the potential for helping quality improvements among other public health programs in Washington and elsewhere.

## Data Availability Statement

The raw data supporting the conclusions of this article, include facilitation plans, meeting minutes, flow charts, reports, presentations, and communications pertaining to the state's process. Some of these materials are available and evident in the article's Supplementary Material. Other related materials can be made available by the authors. Requests to access these datasets should be directed to the corresponding author.

## Author Contributions

SW helped in the initial report writing of this document and contributed to editing and fact-checking. MR was on the project team and was instrumental in creating this work and also held editing responsibility. DH was on the project team and provided feedback and editing. BB led the publication process, provided substantial writing contributions, and heavily edited all versions. All authors contributed to the article and approved the submitted version.

## Conflict of Interest

The authors declare that the research was conducted in the absence of any commercial or financial relationships that could be construed as a potential conflict of interest.
