# Supplementary material for: The Diamond Project: A Quality Improvement Model for Adopting Shared Service Delivery in the Washington Vaccines for Children Program
Source: Front Public Health. 2020 Jul 14;8:272. doi: 10.3389/fpubh.2020.00272 (PMC7372928; doi:10.3389/fpubh.2020.00272)
Supplement: Supplementary file 1 [file Data_Sheet_1.PDF]

Appendix A – Communication Plan

Diamond Project Communication Plan

Situation Analysis

The Washington State Department of Health (DOH) partners with local health jurisdictions (LHJs) to administer several of the grant requirements for the Centers for Disease Control and Prevention (CDC)'s federal Immunization Program Grant through their consolidated contract system. These Vaccines for Children (VFC) grant requirements focus on compliance activities and supporting health provider offices to meet the CDC's immunization program requirements.

In the past few years, an increasing number of LHJs have dropped out of one or more of the seven VFC Tasks within the program and turned their portion of the grant funding back to DOH. In addition, LHJs and DOH began discussing how they might increase their work to improve vaccination rates across the state, which has generated excitement and interest at both the state and local levels.

DOH and the Washington State Association of Local Public Health Officials (WSALPHO) jointly sponsored a cross-jurisdictional improvement project, which began using a lean six sigma approach in March 2016. By updating and improving existing activities through a cross-jurisdictional improvement project, the project team hopes to achieve cost efficiencies, which would enable DOH to reallocate potential savings to immunization promotion activities rather than compliance activities.

There are five project phases:

- Phase 1—Assessment
- Phase 2—Planning & Setting Priorities
- Phase 3—Quality Planning Cycles
- Phase 4—Systems Check and Synthesis of Tasks
- Phase 5—Implementation

The project team completed initial work guided by an initial facilitator, including interviews with LHJ staff to assess the current situation, high level process flow for all seven VFC Tasks, orientation to quality improvement methodology and preliminary analysis of wastes associated with the seven Tasks. When the initial facilitator resigned the position, DOH contracted with the Public Health Centers for Excellence (PHCFE) to resume the project in August. PHCFE facilitated a process for the Diamond Project team at their October 2016 in-person workshop to develop this draft communication plan.

Project goals include:

1. Create a consistent statewide standard of immunization promotion and vaccine compliance services that meets customer needs;
2. Create efficiencies in the services DOH/LHJ provide through consolidated contracts in Washington State; and
3. Identify effective immunization promotion activities to improve immunization rates in Washington State.

Risk Analysis (SWOT)

The Diamond Project team conducted a Strengths-Weaknesses-Opportunities-Threats (SWOT) analysis to better understand the project's strengths and weaknesses (resources and experience), identify opportunities and acknowledge the threats to the success of the project.

Appendix A – Communication Plan

Strengths

- DOH and LHJs are taking a collaborative approach to this project; a community of voices are at the table.
- The project follows a quality cycle that includes conducting a voice-of-the-customer assessment, identifying waste, standardizing processes and using data and evidence-based approaches to guide their work and recommendations.
- The project team composition includes the right people doing the right work.
- The project team shares best practices.

Weaknesses

- Using a shared services approach to this work may lead to loss of personalization with health providers.
- It is difficult for the really small LHJs to participate in every aspect of the project team's work (e.g., only one was able to attend the three-day in-person workshop in Oct.).

Opportunities

- The project may enable LHJs and/or DOH to shift work more toward increasing immunization rates.
- The project will enable the state to modernize its system and process of VFC compliance.
- The project allows WSALPHO to test a CJS approach, which may be able to leverage innovation or other funding.
- A successful project will retire a broken system/process that people are frustrated with.
- An improved system will build new and different relationships (e.g., between LHJs and DOH, among health providers and LHJs, etc.) in a positive way, which in turn could have a positive effect on patient outcomes.
- Washington State would lead the nation in this type of initiative.
- Exposure to this process has taught project team members how to think in a systematic way; this project starts to build a quality infrastructure by training team members on quality concepts, tools and approaches.
- Team members are very enthusiastic about the project's potential.

Threats

- LHJ leaders and immunization program staff fear the loss of funding, jobs, control/autonomy and relationships with local health providers if the Tasks shift from the local level to a shared services or cross-jurisdictional model.
- Some LHJs have already refused to follow this process.
- Not all LHJ immunization program coordinators will go through the full quality process and may not share the same level of enthusiasm as the project team members.

Communication Goals

1. Promote the Diamond Project's aim to decrease waste and increase efficiency so that resources can potentially be redirected to increase immunization rates in the state.
2. Gain support among LHJ leadership and immunization coordinators for the project team's recommendations.
3. Build interest in and excitement for the Diamond Project among key stakeholders.

Key Audiences

Appendix A – Communication Plan

- LHJs
  - Leadership/administrators
  - Immunization program managers and staff
  - Small, rural LHJs
- Washington State DOH
  - Leadership
  - Immunization program staff
- WSALPHO
  - Executive Team
  - Executive Leadership Committee (overlap with LHJ leadership/administrators)
- Health providers enrolled in the VFC program
- CDC

Communication Objectives

LHJs/WSALPHO

- Increase acceptance among LHJ leadership, LHJ immunization coordinators and WSALPHO of a cross-jurisdictional sharing approach to VFC compliance activities.
- Increase LHJ/WSALPHO understanding of how new cost efficiencies in VFC through standardization and decreased wastes could lead to increased immunization promotion work.
- Increase LHJ leadership/WSALPHO's confidence that decisions are based on data, objective criteria, etc.
- Increase LHJ leadership/WSALPHO's understanding how this project aligns with and supports other major public health efforts in the state, e.g., health system transformation.
- Increase project team members' skills and knowledge of quality methods and tools.

DOH

- Increase acceptance among DOH leadership of a cross-jurisdictional sharing approach to VFC compliance activities.
- Increase DOH leadership's understanding of how new cost efficiencies in VFC through standardization and decreased wastes could lead to increased immunization promotion work.
- Increase DOH leadership's understanding how this project aligns with and supports other major public health efforts in the state, e.g., health system transformation.

Health Providers

- Strengthen LHJ and DOH relationships with health providers who participate in the VFC program.

CDC

- Build confidence and interest among CDC funders/decision-makers with the quality improvement and CJS approaches used in this project.

Key Messages

- *We must change how we conduct VFC compliance activities; the current state is not workable.*
  - *Many LHJs have already stopped doing VFC compliance activities because there is not enough money to cover their costs.*
  - *Customer input tells us some of the work is redundant and inefficient.*
- *Changes will result in better health for our communities.*
  - *The potential to focus more on increasing immunization rates excites team members.*

Appendix A – Communication Plan

- *This project is a collaborative effort between LHJs and DOH that includes a lot of immunization expertise at the table.*
    - *LHJs and DOH worked collaboratively to develop draft recommendations for the seven VFC tasks for 2018 and beyond.*
    - *Team members work hard to obtain consensus; they are able to put any differences aside to see the full potential of making positive changes.*
    - *This is an iterative process with several opportunities for feedback.*
- *The project team is following a quality process using customer (LHJ and health provider) input, data, best practices and sound decision-making criteria.*
    - *Team members develop solid criteria and use data to make objective decisions.*
    - *The project follows five phases of a quality planning and improvement project through Dec 2017.*
