# Supplementary material for: The Diamond Project: A Quality Improvement Model for Adopting Shared Service Delivery in the Washington Vaccines for Children Program
Source: Front Public Health. 2020 Jul 14;8:272. doi: 10.3389/fpubh.2020.00272 (PMC7372928; doi:10.3389/fpubh.2020.00272)
Supplement: Supplementary file 2 [file Data_Sheet_2.PDF]

Appendix B -- Delivery Models with Associated Criteria

(1) Centralized Services Criteria Set**:** criteria that defines services best done centrally (state level).

- Public health services that require highly **specialized expertise.**
- Public health services/work that can be done completely **remotely.**
- Public health services that would benefit from **economies of scale.**
- Services associated with **high overhead.**
- When there is a high need for **standardization.**
- Services that could be **effectively** provided centrally according to LHJ Leadership input (from the needs assessment survey conducted).

(2) De-centralized Services Criteria Set**:** criteria that defines services best done de-centrally (by each LHJ).

- When there is a need for LHJs to maintain **local authority** related to the public health service.
- When extensive **knowledge of the local community** is needed.
- When the service requires **geographic proximity.**
- When there is a need to **specialize/tailor the intervention/service.**
- When delivering the service depends on close **community relationships and partnerships.**
- Services that could be **effectively** provided de-centrally according to LHJ Leadership input (from the needs assessment survey conducted).

(3) Cross-jurisdictional Sharing Criteria Set: criteria that defines services could be best done collaboratively across LHJs.

- When LHJs have **shared priorities/common goals**.
- When it makes **geographical sense/proximity**.
- When there are local and regional opportunities to **leverage partnership** (e.g. ACH work).
- When local **cross-jurisdictional expertise** is needed.
- Public health services that would benefit from **economies of scale**.
- Services that could be **effectively** provided via cross-jurisdictional sharing according to LHJ Leadership input (from the needs assessment survey conducted).
