# Supplementary material for: The Diamond Project: A Quality Improvement Model for Adopting Shared Service Delivery in the Washington Vaccines for Children Program
Source: Front Public Health. 2020 Jul 14;8:272. doi: 10.3389/fpubh.2020.00272 (PMC7372928; doi:10.3389/fpubh.2020.00272)
Supplement: Supplementary file 3 [file Data_Sheet_3.PDF]

Appendix C—High-level Flow Chart for Task 6

Sub-Project Definition and Flow Chart for Task 6

**Diamond Project:**<br>Task 6: VFC Site Visits	**Sponsor**	**Task Process Owner**

Description (purpose and background):

Currently, multiple LHJs (8 out of 35) have opted out of providing Site Visits, and processes across those continuing to do Site Visits are highly variable. The Diamond Project team, with support of project sponsors, has agreed to transition Site Visits to a cross-jurisdictional sharing/service (CJS) approach. By developing a CJS model and redesigning and standardizing Site Visit processes across CJS sites, there is potential to realize efficiencies and cost savings that could be redirected to activities that increase immunization rates.

Mission /AIM Statement:

1. **Design** a CJS approach to conducting Site Visits that maximizes efficiencies and minimizes overall program costs.
2. Using re-designed processes, **reduce** the average time per Site Visit, contributing to overall efficiencies and cost reductions in the federal Immunization Program Grant implementation for Washington State.

**Process Measure(s) of Success:**	Outcome Measures:
Process Measures<br>▪ Average time (in minutes) per site visit<br>▪ Average # incidents noncompliance per site visit	▪ Average $ per site visit<br>▪ Average $ training costs/site reviewer

Project Scope

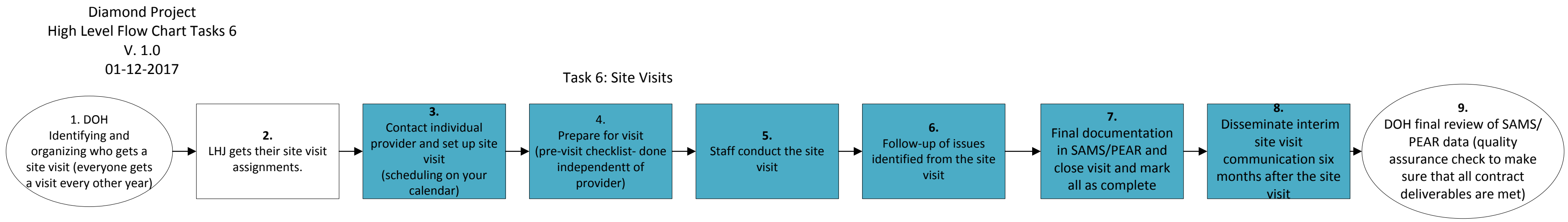

Describe project boundaries, "inclusions"(in-scope) and "exclusions" (out-of-scope)

Constraints/Program Rules:

Site visits must be conducted of all participating providers every 24 months.
