# Supplementary figures and images for: The Diamond Project: A Quality Improvement Model for Adopting Shared Service Delivery in the Washington Vaccines for Children Program
Source: Front Public Health. 2020 Jul 14;8:272. doi: 10.3389/fpubh.2020.00272 (PMC7372928; doi:10.3389/fpubh.2020.00272)

Appendix D--Detailed Process Flow Chart for Task 7

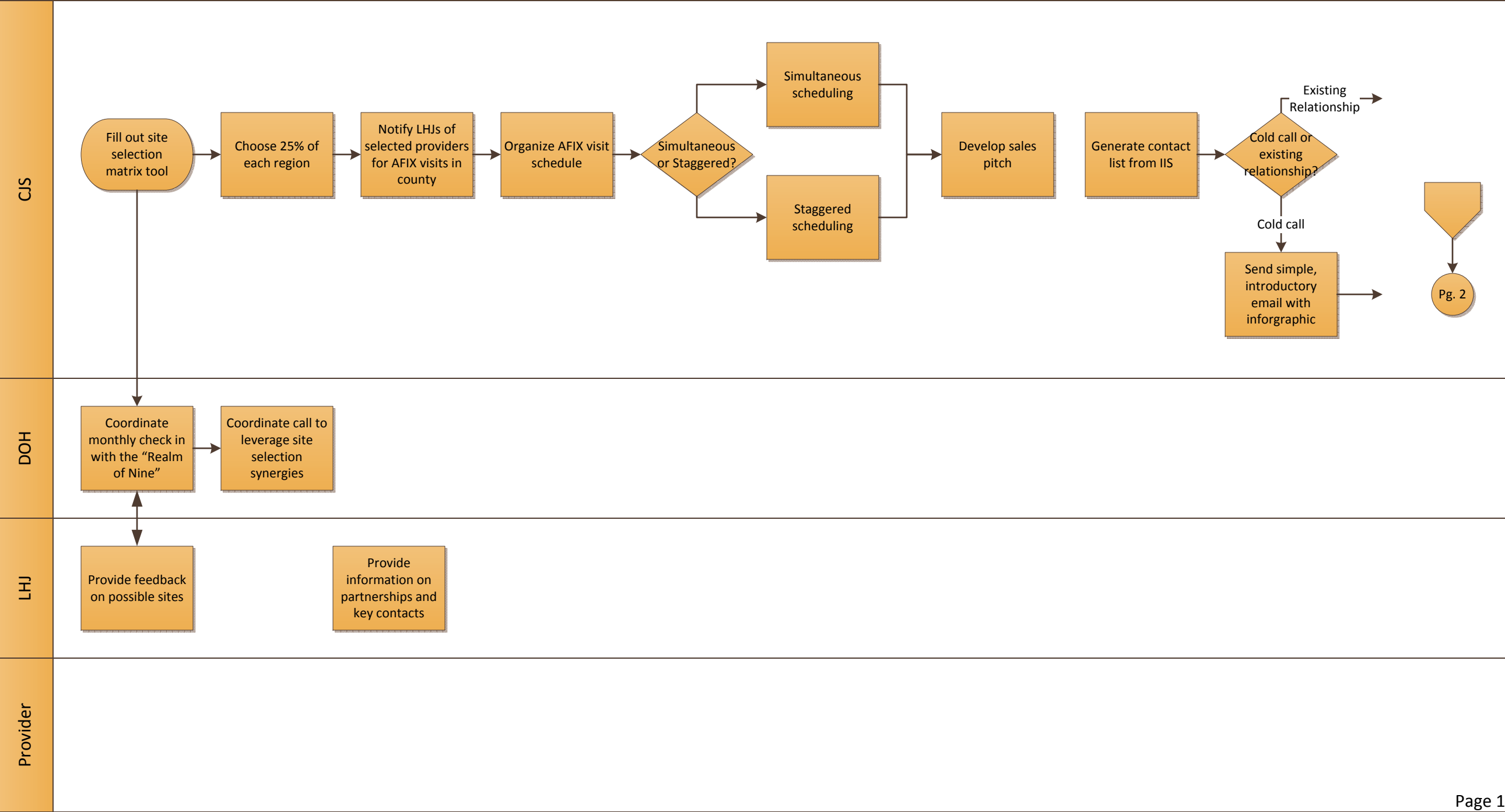

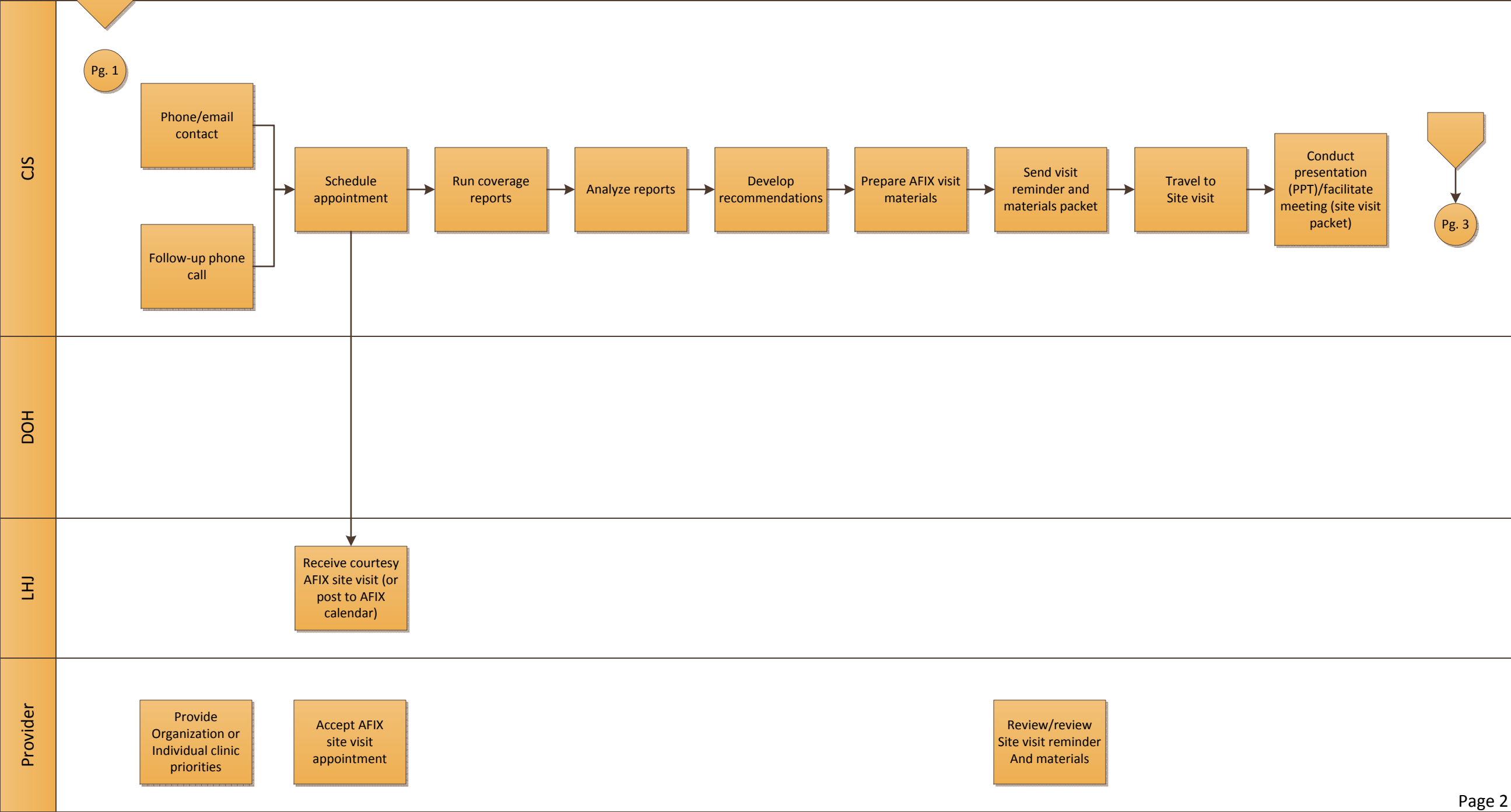

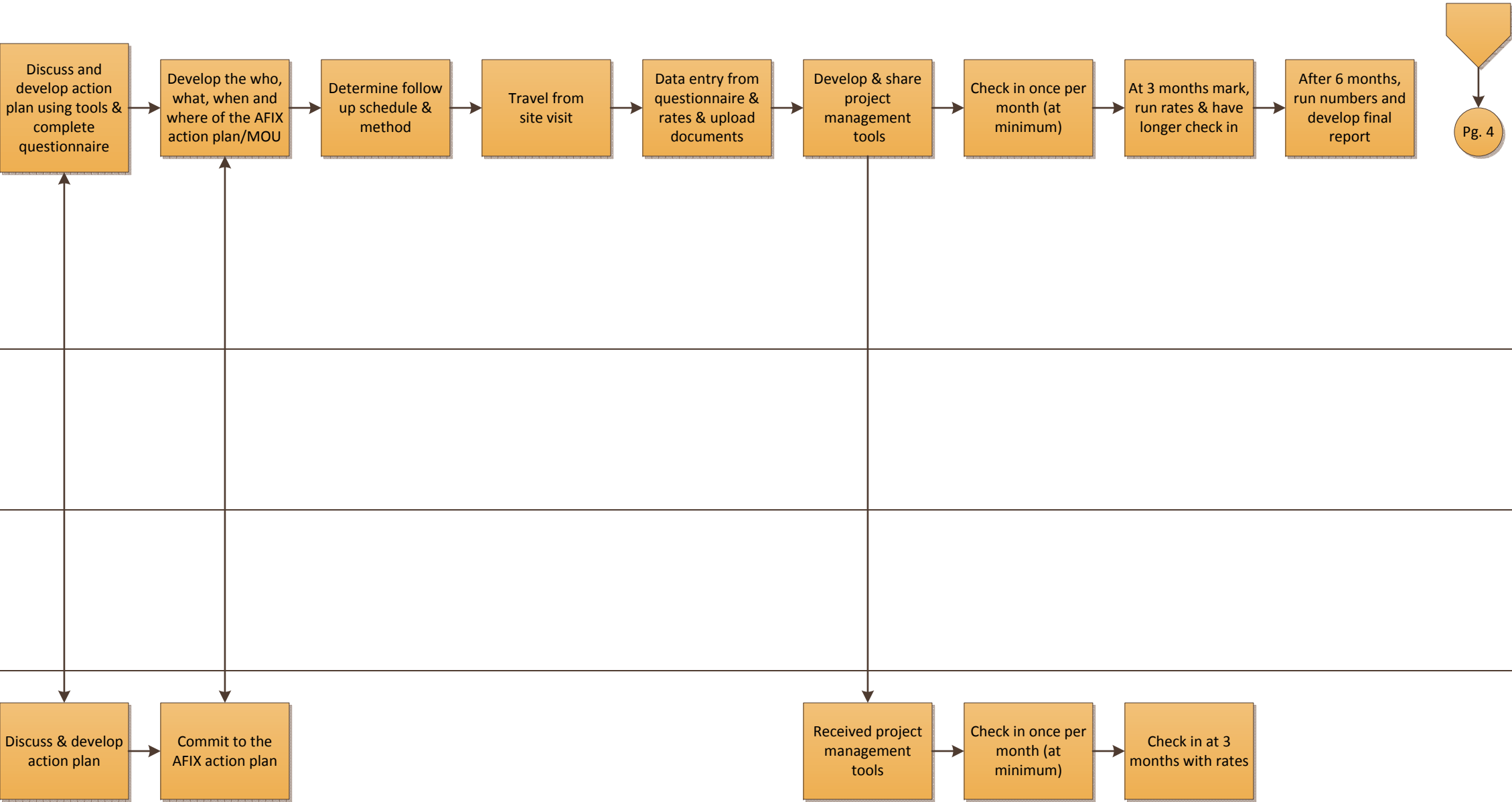

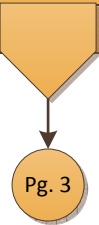

CJS

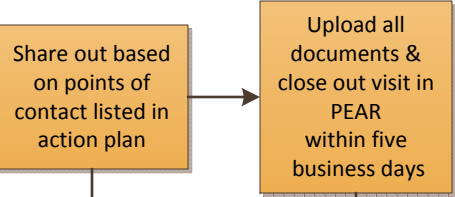

DOH

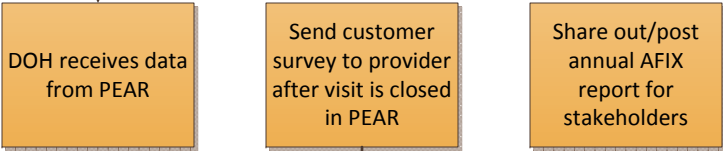

LHU

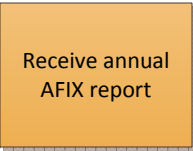

Provider

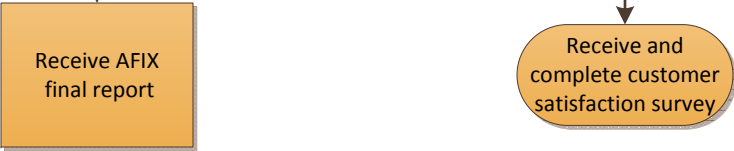

Supplement: Supplementary file 4 [file Data_Sheet_4.PDF]
